# Investigation of the Flexural Behavior of Preloaded and Pre-Cracked Reinforced Concrete Beams Strengthened with CFRP Plates

**DOI:** 10.3390/ma16010022

**Published:** 2022-12-20

**Authors:** S. M. Samindi M. K. Samarakoon, Bartosz Piatek, G. H. M. J. Subashi De Silva

**Affiliations:** 1 Department of Mechanical and Structural Engineering and Materials Science, Faculty of Science and Technology, University of Stavanger, 4021 Stavanger, Norway; 2 Department of Roads and Bridges, Rzeszow University of Technology, 35-959 Rzeszów, Poland; 3 Department of Civil and Environmental Engineering, University of Ruhuna, Galle 8100, Sri Lanka

**Keywords:** CFRP, flexural strengthening, preload, ultimate moment, cracks, reinforced concrete

## Abstract

This paper investigates the flexural behavior of preloaded reinforced concrete (RC) beams, strengthened with Carbon Fiber Reinforced Polymer (CFRP) plates using an experimental program, analytical procedure, and Finite Element Method (FEM) simulation. The RC beams were subjected to preloads of 30%, 50% and 70% of the yielding load, prior to installation of the strengthening system. The eight RC-strengthened beams with a reinforcement configuration of 3Ø12 and two CarboDur S512 plates have been evaluated using bending tests. The failure modes of all the RC-strengthened beams were governed by the widening of flexural cracks within a constant bending zone, followed by debonding of the CFRP plates. The plates were debonding simultaneously or one plate prior to the other plate. The ultimate moment capacity is not significantly reduced while increasing preload levels from 0% to 70%. The moment capacity is increased by 70% to 80% in the CFRP strengthened beams, compared with un-strengthened beams indicating the potential of capacity enhancement that can be attained by externally bonded CFRP.

## 1. Introduction

The strengthening of reinforced concrete (RC) structures has recently become an increasingly important issue for both new and old structures due to degradation, construction deficiencies, loss of structural serviceability and sudden damages, possibly, caused by natural disasters, wars, and earthquakes [1,2]. In reality, structural members are subjected to a load history, preloading [3,4] or damage (e.g., cracks) [5] prior to strengthening. Load history, preloading or prior damage have been considered in recent studies [6,7,8,9,10,11,12,13] but no consideration has been given for preloading with different durations, extreme magnitudes, and its effect on the flexural behavior of RC beams strengthened with CFRP plates. RC beams with externally bonded CFRP have been tested after the application of preloading of 30% of the nominal capacity [7,8,9], 35% of design load [10], 40% of steel yielding [11,12], 60–66% of steel yielding [9,10,12] and 80–100% of steel yielding/flexural yielding load [9,11,13]. Moreover, most of the studies on extreme preloading conditions have been limited to numerical investigations and a large number of experimental studies are an imperative need, in order to understand the actual performances of preloaded and pre-cracked reinforced concrete beams with externally bonded CFRP plates. To stimulate the effect of preloading during an experiment, the beams have been preloaded to pre-defined loads before, after or during strengthening [9] and no attention has been given for preloading duration and its effect on the flexural behavior of strengthened RC beams or RC beams strengthened with CFRP plates.

The effect of preloading on the ultimate carrying capacity of RC beams is a primary concern in most experimental programs [6,14]. In preloaded beams, compared with control beams, there were significant improvements in flexural capacity: ranging from 28% to 102% [14,15] whereas, there were insignificant improvements in ultimate load capacity [7,11,16], poor strengthening performance [9] or flexural capacity decreased as preload increased [16]. Due to this inconsistency, there is a growing need for further research on experimental and numerical investigations to verify at which preload level the strengthening performance is affected. In addition, most of the previous experimental testing programs were limited to similar reinforcement configurations (i.e., two steel reinforcements at the base of the cross section) in RC cross sections with different types of CRFP plates, and no attempts have been given for different reinforcement configurations.

It is vital to pay attention to possible improvements of the concrete-FRP interface because debonding is the main failure mode. There are seven potential failure modes which have been mostly observed during the testing of RC beams with FRP sheets, irrespective of their preloading condition [17]. During the testing of preloaded beams, it has been often observed occurring of similar failure modes: concrete crushing [11], rupture of CFRP plates [9,11,18] and debonding [8,9,10,12,13,19]. However, during the testing of flexural-strengthened RC beams with CFRP plates, the most observed failure mode is debonding, which can be plate end debonding, intermediate flexural crack induced interfacial deboning and intermediate flexural-shear crack induced interfacial debonding [17]. Nevertheless, as the flexural strengthened RC beams failed suddenly, it is challenging to trace the governing debonding failure mode of flexural-strengthened RC beams with CFRP plates.

The cracking behavior of preloaded beams is an important aspect when setting verifications at serviceability limit states. Many researchers have studied cracking behavior at the initial cracking stage, post cracking stage and post-yielding stage and discussed the variation of tangent stiffness and flexural stiffness. Jian-He et al. [19] found that RC beams without a preloaded condition had an elastic phase before cracking and slightly higher flexural stiffness, compared with that of preloaded beams. In addition, it has been found that the average tangent stiffness of preloaded RC beams can be higher than that of beams without preloading during both post-cracking and post-yielding stages [7]. However, there are limited studies on crack width and crack spacing at the stabilized cracking stage.

The objective of the current study is to investigate the effect of preloading conditions (magnitude, duration) on the flexural behavior of CFRP strengthened RC beams with a reinforcement configuration of three bars of Ø12 mm, installed at the base of the section using a comprehensive experimental program, analytical procedure and Finite Element Method (FEM) simulation. Eight beams of 250 mm × 300 mm cross section and 2200 mm length were cast at an average cylindrical concrete compressive strength of 49 MPa (i.e., 28 days). The preload of 30%, 50% and 70% of yield load of un-strengthened RC beams were chosen to reflect the occurrence of initial cracks, flexural cracks and flexural-shear cracks. Cracking behavior, ultimate moment capacity, failure modes, tangent stiffness and strain development in the CFRP plates during loading of the preloaded RC beams were compared to the beams without preloading. Moreover, the theoretical bending moment capacity was estimated and compared with the experimental moment capacity. A non-linear FEM simulation was carried out, verifying the experimental findings to determine the failure mechanism and the potential of capacity enhancement that can be attained with externally bonded CFRP.

## 2. Materials and Methods

### 2.1. Preparation of Reinforced Concrete Beams

**Figure 1:**
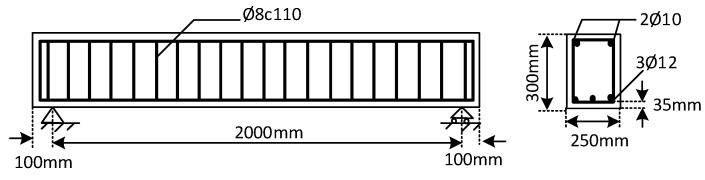
Geometry and steel reinforcement of the beams.

Twelve RC beams were casted, with the reinforcement configurations shown in Figure 1, to study the effect of preloading on the flexural behavior of RC beams. All the casting and testing procedures were carried out at University of Stavanger, Norway. Four of the beams were used to estimate the yielding load of the un-strengthened beams. All of the beams were designed with a cross sectional dimension of 250 mm × 300 mm, and a total length of 2200 mm. The longitudinal reinforcement consisted of two bars of Ø10 mm, installed on the top, and three bars of Ø12 mm, installed at the base of the section. The stirrups were bars of Ø8 mm, placed at 110 mm center to center spacing, as shown in Figure 1. Twelve beams were casted using two batches of ready mixed concrete mixtures (A and B). The composition of the mixtures that received from the ready mixed concrete supplier is given in Table 1. Two beams from each batch were used to implement conditions without preloading, as well as with preloading, of 30%, 50% and 70% of the yielding load of the unstrengthen beams (Py).

### 2.2. Properties of Materials

#### 2.2.1. Concrete and Steel Reinforcement

The average cylindrical compressive strength of concrete at 28 days was 51 MPa in Batch A and 47.5 MPa in Batch B. The average splitting tensile strength of the concrete at 28 days was about 3.3 MPa in both Batch A and Batch B. All steel reinforcement had a characteristic yield strength of 500 MPa and modulus of elasticity of 200 GPa.

#### 2.2.2. CFRP Plates (CarboDur S512) and Epoxy Adhesive

The mechanical properties of CFRP plates (width × thickness × length: 50 mm × 1.2 mm × 2000 mm) is given in Table 2. Eight of the 12 beams were strengthened by pasting two CFRP plates together. To bond the CFRP plates on the surface of the RC beams, the epoxy adhesive Sikadur^®^-30 was used. The epoxy adhesive is based on a combination of epoxy resins and special filler, designed for use at normal temperatures between +8 °C and +35 °C [14] and the properties are given in Table 3.

### 2.3. Test Set Up and Instrumentation

**Figure 2:**
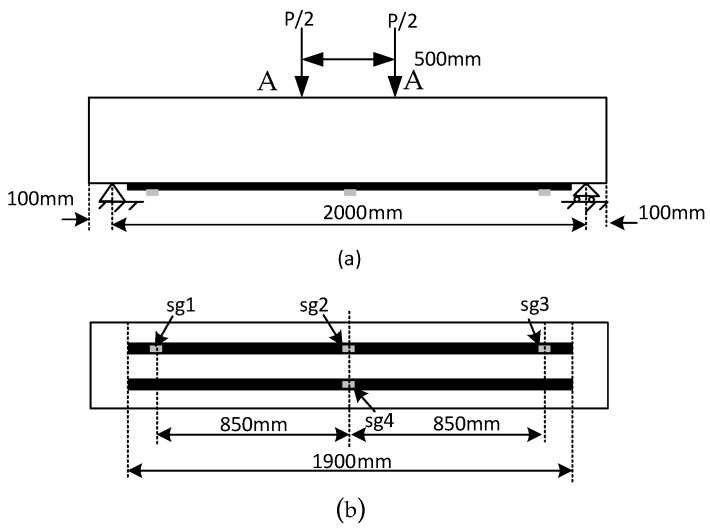
(**a**) Experimental set up (**b**) location of strain gauges on the bottom face of the beams.

All of the beams were tested under a four-point bending test setup with a constant bending region of 500 mm and the span length between two supports was 2000 mm. The load was transferred to the RC beams at a 10 kN/min loading rate via a hydraulic jack attached to the test setup of ‘Toni Technik’ apparatus. The apparatus was manufactured by Toni Technik, Berlin, Germany. The applied load and deflection were monitored during the preloading and during the flexural test until beam failure occurred. The deflection was taken from the apparatus, and it was monitored at the load applications points as given in “A”. Strain gauges (sg1, sg2, sg3 and sg4) were mounted over the surface of the CFRP plate to study the strain development in preloaded beams and in beams without a preloaded condition, as shown in Figure 2.

### 2.4. Testing Procedure

**Figure 3:**
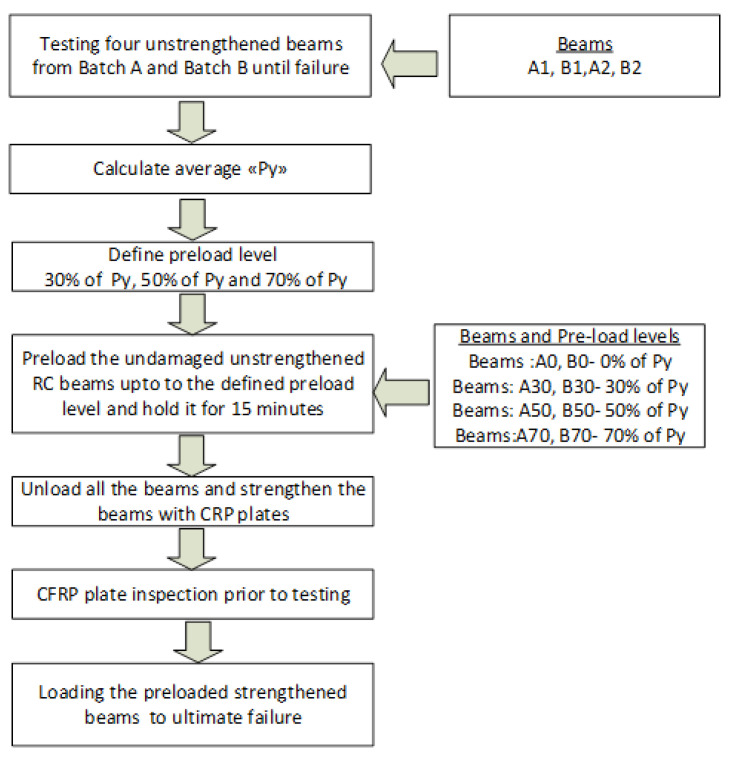
Testing Procedure.

**Figure 4:**
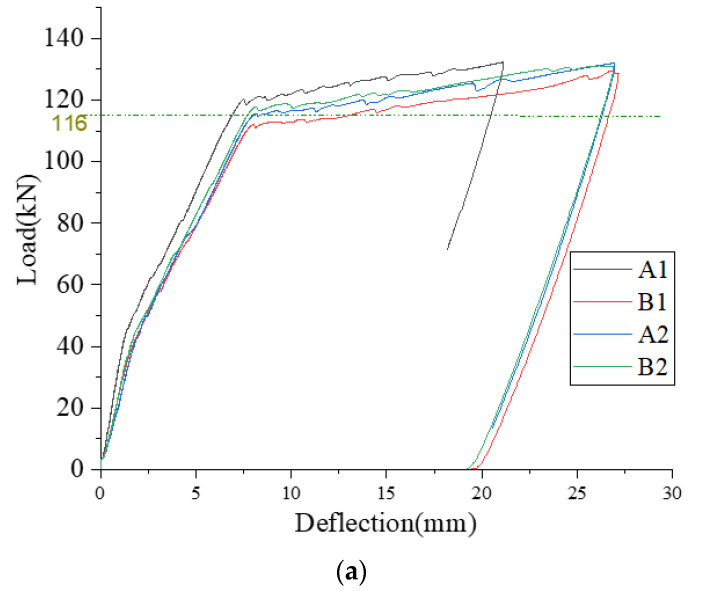

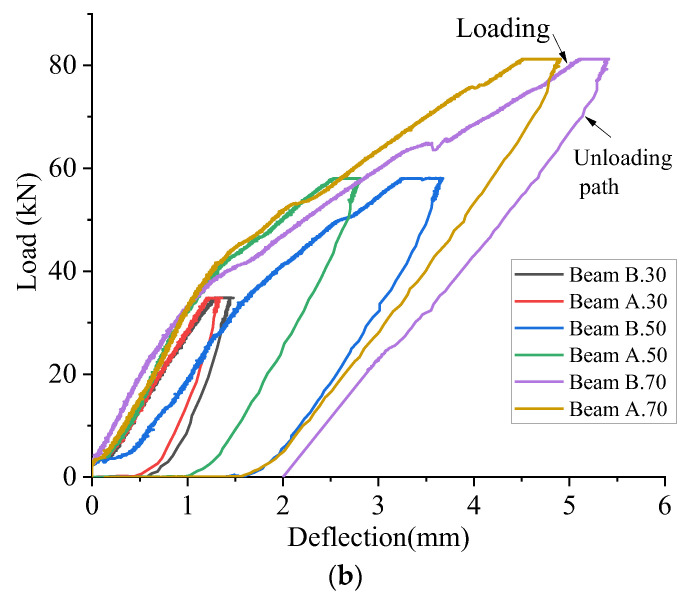
(**a**) Load vs. deflection behavior of un-strengthened beams, (**b**) Load vs. deflection behavior during preloading of beams.

The testing procedure shown in Figure 3 was used for this study. Initially, four randomly selected, un-strengthened beams from Batch A (A1, A2 beams) and Batch B (B1, B2 beams) were subjected to four-point bending tests to find the average yield load (Py). The average ‘Py’ was found to be 116 kN, as shown in the load vs. deflection curves in Figure 4a. The preload levels were defined as 30% of Py (34.8 kN), 50% of Py (58 kN) and 70% of Py (81.2 kN). The preloading levels were chosen based on the damage level classification given in Choi and Yun [22], to reflect initial cracking, flexural cracks and flexural-shear. After defining preloading levels, two un-strengthened beams from each batch were given the aforementioned preloading levels and held for 15 min. The load vs. deflection behavior during preloading and unloading is shown in Figure 4b.

#### Crack Formation during Preloading Prior to Pasting CFRP Plates

**Figure 5:**
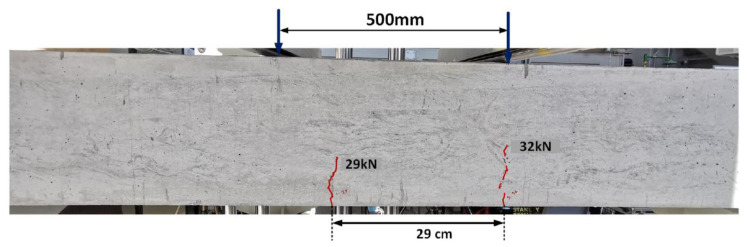
Crack formed in the B.30 beam (30% of Py).

Two batches of ready mixed concrete were used for the casting of the beams (Batch A and Batch B). A.30 (from Batch A) and B.30 (from Batch B) were preloaded to 34.8 kN, as shown in Figure 4b. The first crack was observed at 29 kN during preloading of the B.30 and at 30 kN in A.30 beams. Two flexural cracks within the constant bending zone were observed in both beams. Figure 5 shows the cracks that formed at 29 kN and 32 kN in Beam B.30. Although both beams had the same configuration, there was some variability in crack spacing. The crack spacing was 290 mm and 370 mm in the A.30 and B.30 beams, respectively.

**Figure 6:**
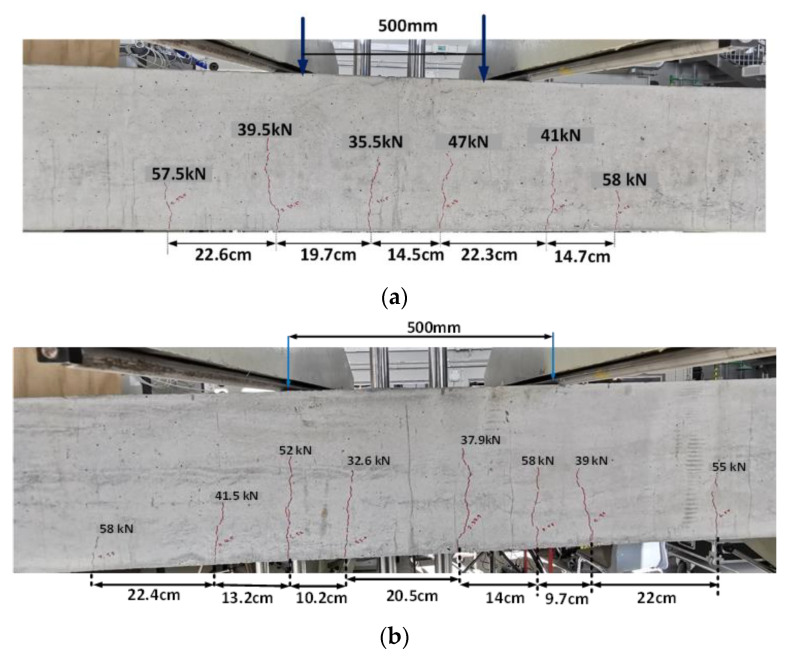
(**a**) Beam A.50 (50% of Py). (**b**) Beam B.50 (50% of Py).

Beam A.50 (from Batch A) and Beam B.50 (from Batch B) were preloaded to 58 kN (50% of Py (56 kN)). The flexural cracks in Beam A.50 are shown in Figure 6a. Within the constant bending zone, three to four cracks were observed in both beams. However, two flexural-shear cracks appeared in Beam B.50. There was no significant difference between the maximum crack spacing in the A.50 and B.50 beams within the constant bending zone (see Figure 6a,b).

**Figure 7:**
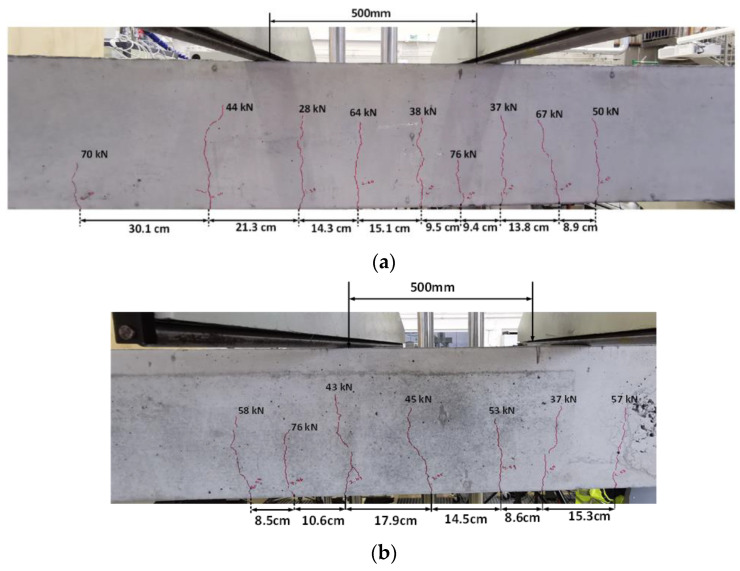
(**a**) Beam B.70 (70% of Py). (**b**) Beam A.70 (70% of Py).

Beam A.70 (from Batch A) and Beam B.70 (from Batch B) were preloaded to 81.2 kN (70% of Py (81.2 kN)). Figure 7a shows the flexural cracks observed in Beam B.70. Between nine and eleven cracks were visually observed in Beam A.70 and B.70, respectively. Within the constant bending zone, four to five flexural cracks were observed in both beams. Four to five flexural-shear cracks appeared in the constant bending zone. Within the constant bending zone, there was no significant difference the maximum crack spacing of A.70 and B.70 (see Figure 7a,b), within the constant bending zone.

### 2.5. CFRP Plate Inspection Prior to Testing

The cracks initiated during preloading and closed when the load was removed. Hence, no substantial cracks were present during the installation of the strengthened system. The application of the CFRP plates were performed in accordance with the installation procedure described in the method statement for the Sika CarboDur system [23]. Initially, the bottom surfaces of all the beams were leveled and prepared using a concrete grinder. The bottom surfaces were brushed, vacuumed, and wiped clean with acetone to remove any dust or rest from formwork oil [24]. The CFRP plates were attached on an approximately plane substrate. To allow for development of the full design strength of the adhesive, the strengthen beams were cured for minimum 7 days at 20 °C. A visual inspection was carried out to examine the bond quality of the strengthened beams before starting the four-point bending test. Table 3 summarizes the results of the visual bond inspection and the bond defects detected. According to the inspection results given in Table 4, a good bond condition was observed between concrete and CFRP plates in most of the strengthened beams, with uniform thickness and hardened edges of the adhesive on both sides of the plates. However, higher number of voids between the plate and the concrete were found in A.50 beam indicating poor bond quality. There are limited discussions in the literature about the inspection procedure of bond conditions prior to the testing of strengthened beams in laboratories. A simple method was used to determine the extent of the voids, by gently tapping on the CFRP plates and using the resonating sound as an indicator to identify whether it was fully bonded or if there were voids within the adhesive layer [24]. The approximate depth of the bond defect (d) was evaluated using a thin steel wire inserted between the CFRP plate and the concrete.

## 3. Discussion of the Results of the Experiment

### 3.1. Cracking Behavior

**Figure 8:**
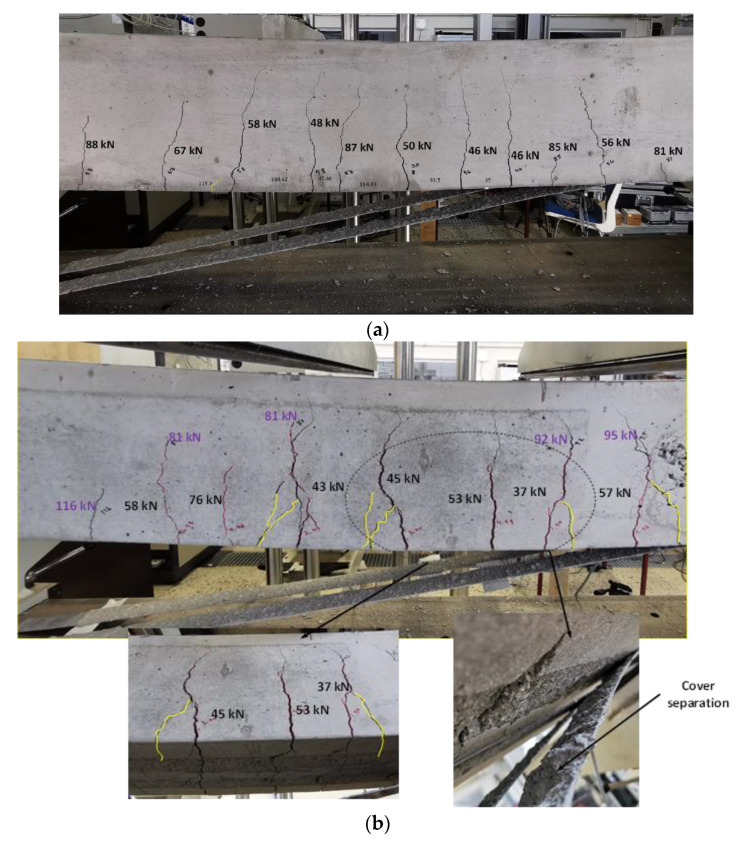
(**a**) B.0 beam (0% of Py). (**b**) A.70 beam (70% of Py).

The strengthen beams were tested after around 20 days from preloading. The crack formation during reloading of the B.0 and A.70 beams are shown in Figure 8a,b (cracks during preloading are marked in red and the reopened cracks and new cracks formed during reloading are marked in black). The first cracks appeared in both strengthened beams without preloading (i.e., A.0 and B.0) at 50–52 kN whereas, the reopened cracks that formed during preloading, were observed in the strengthened and preloaded beams before reaching 50 kN. This confirms that CFRP plates delayed the formation of the first crack in strengthened beams without a preload condition compared to preloaded beams. Ten to eleven cracks (flexural and flexural-shear cracks) appeared in the A.0 and B.0 beams. The A.30 and B.30 (30% preload) beams had 13 to 14 cracks, the A.50 and B.50 (50% preload) beams had 10 to 12 cracks and the A.70 and B.70 (70% preload) beams had 10 to 13 cracks. The results show that when the preload level is 30%, a higher number of cracks were observed compared to other preload levels. It could be seen that there is no correlation between the number of cracks observed vs. preload levels.

### 3.2. Failure Modes during Four-Point Bending Test

**Figure 9:**
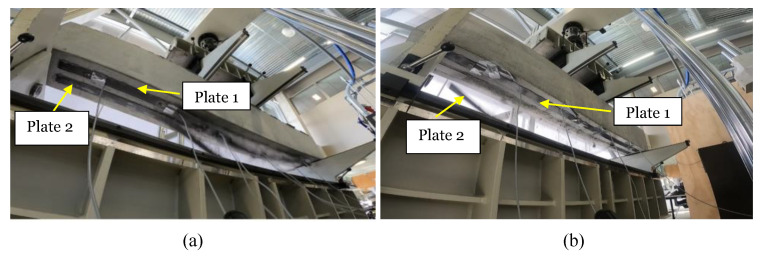
(**a**) Simultaneous failure of two CFRP plates in the B.0 beam; (**b**) failure of one plate before the other in the A.0 beam.

According to the previous literature, plate end interfacial debonding, interfacial debonding induced by an intermediate flexural crack (IFC) or by intermediate flexural shear cracking have been considered as the most common potential failure modes [17,25]. In this study, a GoPro Hero9 speed camera was set up during testing under the strengthened beams, to capture the failure modes. Using the slow motion mode in video editor software, the failure modes have been further studied. It has been observed that no cover separation (i.e., without preload) but substrate removal was observed in B.0 and A.0. It has been observed that the failure of B.0 started with widening of the cracks in the constant bending zone, resulting in the breaking of bonds between two CFRP plates. Both plates delaminated at the same time, leading to the failure of the beam shown in Figure 9a. After reaching the failure load, CFRP plates were delaminated by more than half of the beam length, as shown in Figure 8a. The A.0 beam shows crack opening and delamination of one plate in the constant bending zone and delamination of the other plate after a few milliseconds, as shown in Figure 9b. Moreover, two diagonal cracks (secondary cracks) can be seen, forming at the failure in Beam A.0. Hence, the failure mode of strengthened beams (without preload) is IFC debonding leading to plate end debonding.

In A.30, complete delamination of one plate was observed before delamination of the other plate after a few milliseconds. In addition, diagonal cracks were found after failure. The opening of diagonal cracks was wider than in the strengthened beams without a preload condition. However, in B.30, both plates delaminated at the same time and no diagonal cracks formed during failure. The failure mode is IFC debonding leading to plate end debonding, or complete debonding of the plates.

When the preload level was 50% in the A.50 beam, failure started with widening of the flexural cracks near concentrated loads and delamination in the constant bending zone, leading to delamination at both ends of one plate prior to the other. The delamination of the other plate in the constant bending zone led to end delamination. No secondary cracks were formed. During the visual inspection of the bond condition of the A.50 beam, it was found that poor bonding leads to lower failure loads, compared with the others. In B.50, failure starts by widening cracks and the start of delamination of one plate at the constant bending zone. Moreover, delamination of one plate prior to the other plate can result in secondary cracks formed during failure. In A.70 and B.70, both CFRP delaminated at the same time and wider diagonal cracks formed (diagonal cracks marked in yellow in Figure 8b) than in the 50% preload level. Slight cover separation was observed in the proximity of wide crack opening, when preloading is greater than 50%.

When comparing the beam testing conducted for a preload level at 30% by Zhang, et al. [8] for 2Ø12-reinforcement configuration, the failure mode of the present study gives good agreement with their findings. However, CFRP rupture was not observed during the present study. It is given in literature that during laboratory testing CFRP rupture is a rare event and plate separation due to debonding is more probable [26]. A similar failure mode has been observed during this experiment. In addition, very few studies have been conducted in the existing literature for preload levels of 50% and 70%. Compared with the failure modes observed by Shin and Lee [16] for preload levels of 50% and 70%, where bond failure between concrete and CFRP sheets occurred, the present study shows similar failure modes of IFC debonding and plate end debonding at the level of 50% and 70% preload. In addition, when preload level is 50% and 70%, the slight cover separation observed in the proximity of wide crack opening has not been discussed by previous literature on the effects of preloading (see Figure 8b).

### 3.3. Load vs. Deflection Behavior of Strengthened Beams with and without Preload

**Figure 10:**
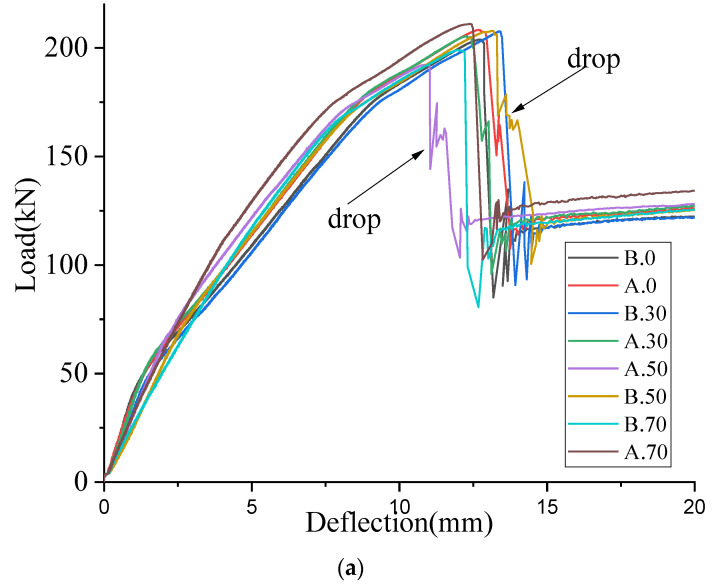

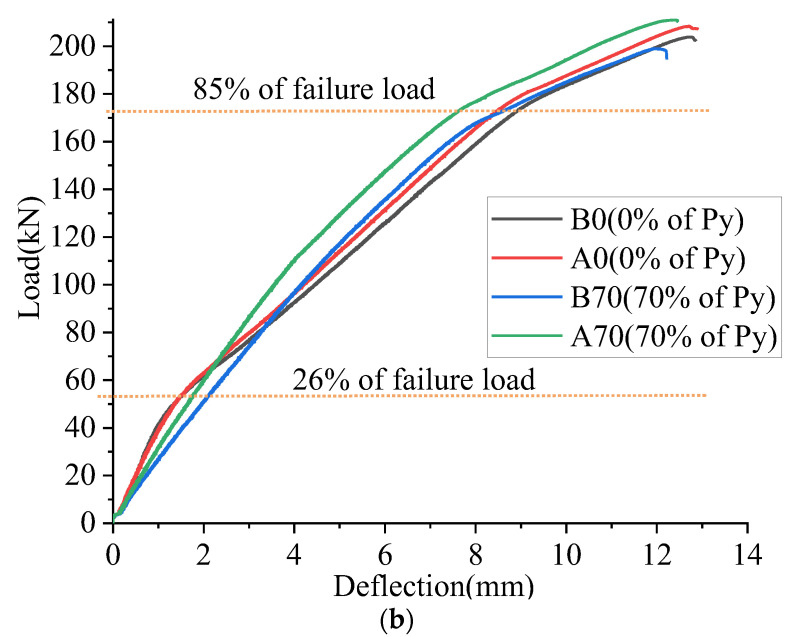
(**a**) load vs. deflection behavior in all strengthened beams, (**b**) load vs. deflection behavior in 0% vs. 70%.

Load vs. deflection curves of all the strengthened beams, with preload and without preload conditions, are given in Figure 10a. The average ultimate load of the strengthened beams, with a preload level of 30% and without preloading, was about 206 kN. However, when the preload level was 50%, the average ultimate load was 200 kN and when the preload level was 70%, the ultimate load was 205 kN. Hence, there is no significant decrease in ultimate failure load when increasing the preload level. It could be observed that a sudden drop of load in beam, A0, A.30, A.50 and B.50 after reaching the ultimate load, as shown in Figure 10a. This is due to the aforementioned delamination of one plate prior to the other.

When comparing the load vs. deflection curves of 0% preloaded strengthened beams vs. 70% preloaded strengthened beams (see Figure 10b), three different gradients have been found in the 0% preload condition but two gradients in the 70% preload. In 0% preload beams, the initial cracking stage, post cracking stage and post-yielding stage can be clearly identified using three gradient changes. The initial gradient change occurs at the load of first cracking with 0% preloading. This is around 26% of the average failure load of strengthened beams without a preload condition (Pmax.0), as given in Figure 10b. The next gradient change has been found at 85% of Pmax.0. Up to 26% of Pmax.0, the tangent stiffness of 70% preloaded strengthened beams is 21–35% less than that of 0% preload beams. This is mainly due to the fact that preloaded beams were cracked before the pasting of the CRFP plates and crack opening during reloading, resulting in reduced tangent stiffness. Between 26% and 80% of Pmax.0, there is a higher tangent stiffness observed in the 70% preloaded beams, compared to the 0% preloaded beams. However, above 80% of Pmax.0, there is no significant difference between the tangent stiffness of 0% preload and 70% preload. Irrespective of preload level, a similar observation has been reported in the literature, by Zhang, et al. [8] and Jian-He, et al. [19].

Similar analysis has been carried out to compare 0% preloaded beams with 30% and 50% preloaded beams. There is no significant difference between the gradient up to 26% of Pmax.0 in both 0% preloaded beams and 30% preloaded beams. This indicates that when the preload level is low, the behavior is quite similar to the 0% preload condition. Nevertheless, two gradients observed in the 50% preload condition were similar to the 70% preload condition. When comparing 0% preloaded, strengthened beams with 50% preloaded beams (up to 26% of Pmax.0), a 17–28% reduction of tangent stiffness was found. This reduction is less than that of the 70% preload condition. Hence, the tangent stiffness reduction significantly increased when the preload level was over 50%. Therefore, it is vital to take into account the stiffness differences occurring in beams with preload and without preload, when setting serviceability limit state verifications.

### 3.4. Experimental Ultimate Bending Moment

The results of the experiment including un-strengthened beams (A1, B1, A2, B2) and strengthened beams with different preload levels are given in Table 5. Although there is a slight decrease in the average ultimate load when increasing the preload level, no significant differences were seen in the average ultimate load between strengthened beams with preload and without preload. The experimental ultimate bending moment (MR.exp) was calculated based on the failure load in the strengthened and un-strengthened beams, as given in Table 5. The percentage increase of MR.exp has been calculated with respect to the average experimental ultimate bending moment of un-strengthened beams. A moment capacity increase between 70 and 80% was found in all beams except A.50, which had low bond quality. The effect of preload level on ultimate bending moment capacity was calculated with respect to an average value of ‘0%_S’. It can be seen that, when preload level increases from 30% to 70%, average ultimate bending decreases by 1–7%. Therefore, this confirms that the decrease in moment capacity, when increasing preload from 0 to 70% is not significant. This finding gives good agreement with previous literature [8,11,18]. In addition, when compared with strengthened beams without a preload condition, the maximum strain at the middle of the beam was decreased by about 7%. However, 50% preloaded beams gave a 20% decrease, due to poor bond condition.

There are practical difficulties when carrying out the experimental program due to the availability of testing facilities that can hold preloading for a long duration. Therefore, 15 min duration was chosen to be the duration of the preload. However, the influence of the preloading duration on the ultimate moment capacity has not been studied. Moreover, both preloading and four point bending testing were carried out using the same testing facility and a permanent loading scenario during installation of the strengthening system could not be maintained. The change in the preloading scenario can result in different test results, if there are permanent initial deformations in the beams.

### 3.5. Comparison of Experimental Ultimate Bending Moment with Theoretical Flexural Capacity

Theoretical values of flexural capacity and deflections were calculated using analytical procedure proposed by Piątek [27] which was developed on the basis of the European standards and guidelines for designing RC structures and their strengthening by FRP materials [28,29,30]. The analytical procedure uses the following assumptions:

- the Euler–Bernoulli theory of plane sections is applicable [28];
- the strain in the reinforcement bonded with concrete, whether in tension or in compression, is the same as that in the surrounding concrete [28];
- the tensile strength of the concrete is neglected [28];
- the parabolic-rectangular distribution of stresses in the concrete in compression is simplified to the form of a rectangle by means of appropriate coefficients of the stress distribution shape according to the [28,29];
- the stresses in the reinforcing steel are determined on the basis of a bilinear stress-strain relation with a horizontal top branch [28];
- the FRP stress-strain relationship is linear until the ultimate strains occur [29];
- three possible failure modes of RC beam strengthened in flexure with the CFRP strips are considered: concrete crushing in compression zone, CFRP strip debonding and CFRP strip rupture;
- the ultimate strains in the FRP material due to the possibility of debonding are determined based on the assumptions of the Italian standard [30];
- the ultimate strains in the FRP material due to the possibility of breaking are determined using the material factor [29] and the strain correlation factor.

A crucial parameter to evaluate flexural capacity is the ultimate strain in the FRP material. In the case of the analyzed beams, strains caused by the strip debonding were calculated according to Equation (1).

(1) $$\Delta \varepsilon_{f}=\frac{k_{cr}}{E_{f}\cdot \gamma_{f}\cdot \sqrt{\gamma_{c}}}\cdot \sqrt{\frac{2\cdot E_{f}\cdot \Gamma_{Fk}}{t_{f}}}$$

where: $\Gamma_{Fk}=0.03\cdot k_{b}\cdot \sqrt{f_{ck}\;f_{ctm}}$—fracture energy for concrete connected with the CFRP strip, $k_{b},\;k_{cr}$—factors according to the [29], $\gamma_{f},\;\gamma_{c}$—material safety factors for composite and concrete (equal to 1.0 for comparison with the experiment), $E_{f},\;t_{f}$—modulus of elasticity and the thickness for CFRP strip.

The depth of the compression area (*x*) is determined iteratively on the basis of the equilibrium equation of horizontal forces and linear distribution of the strain in the cross-section (Equations (2) and (3)). Eventually, flexural capacity of the beam is calculated using equilibrium equation of moments in the cross-section (Equation (4)).

(2) $$\varepsilon_{c}\left(x\right)=-\Delta \varepsilon_{f}\frac{x}{h-x}\;$$

(3) $$A_{f}\;\Delta \varepsilon_{f}\;E_{f}+A_{s1}\;\varepsilon_{s1}\;E_{s}-k_{1}\;x\;b\;f_{ck}-A_{s2\;}\varepsilon_{s2}\;E_{s}=0_{\;}$$

(4) $$M_{R.th}=A_{f}\;\Delta \varepsilon_{f}\;E_{f}\;h+A_{s1}\;\varepsilon_{s1}\;E_{s}\;d_{1}-k_{1}\;x\;b\;f_{ck}\;k_{2}-A_{s2\;}\varepsilon_{s2}\;E_{s}\;d_{2}\;$$

where: *h*, *b*—height and width of the beam, $A_{f},A_{s1},A_{s2}$—cross-sectional area of CFRP strip, top and bottom steel reinforcement, $\varepsilon_{s},\;E_{s}$—steel strains and elastic modulus, $d_{1},\;d_{2}$—depth of top and bottom reinforcement, $f_{ck}$—characteristic concrete compressive strength, $k_{1},\;k_{2}$—factors, characterizing the width of the stress block and the location of the compressive force in the concrete, respectively [29].

Theoretical flexural capacity ($M_{R.th}$) calculated using the Equations (1)–(4) for the analyzed beams was equal to 76.6 kNm. The *M_R_._exp_*/$M_{R.th}$ ratio is estimated as given in Table 5 for all the beams. It can be seen that all the beams give good agreement with the experimental findings irrespective of the preload level.

To calculate the deflections, the analytical model developed by Pellegrino and Modena [31] was adopted, which allows for the definition of the deflection in three working stages of the strengthened reinforced concrete beam (uncracked, post-cracked and post-yielded). To determine the deflection, it is necessary to find ultimate loads for the subsequent stages: the cracking moment ($M_{cr}$), the steel yielding moment ($M_{y}$), and the characteristic ultimate flexural capacity ($M_{u}$). These values are determined on the basis of the linear relation of strains in the cross-section. Then, the curvature of the member is calculated under the loads causing cracking ($\theta_{cr}$), steel yielding ($\theta_{y}$) and failure ($\theta_{u}$), using the Equations (5)–(7).

(5) $$\theta_{cr}=\frac{M_{cr}}{E_{c}\cdot I_{cs}}$$

(6) $$\theta_{y}=\frac{\varepsilon_{slim}}{d-x_{y}}$$

(7) $$\theta_{u}=\frac{\Delta \varepsilon_{f}}{d-x_{n}}$$

where: $M_{cr}$—cracking moment, $E_{c}$—modulus of elasticity of concrete, $I_{cs}$—moment of inertia of uncracked cross-section, $\varepsilon_{slim}$—yielding strain of steel, $\Delta \varepsilon_{f}$—characteristic composite strain increase, d—effective depth of the cross-section, $x_{y},x_{n}$—depth of the compression area at yielding and failure.

**Figure 11:**
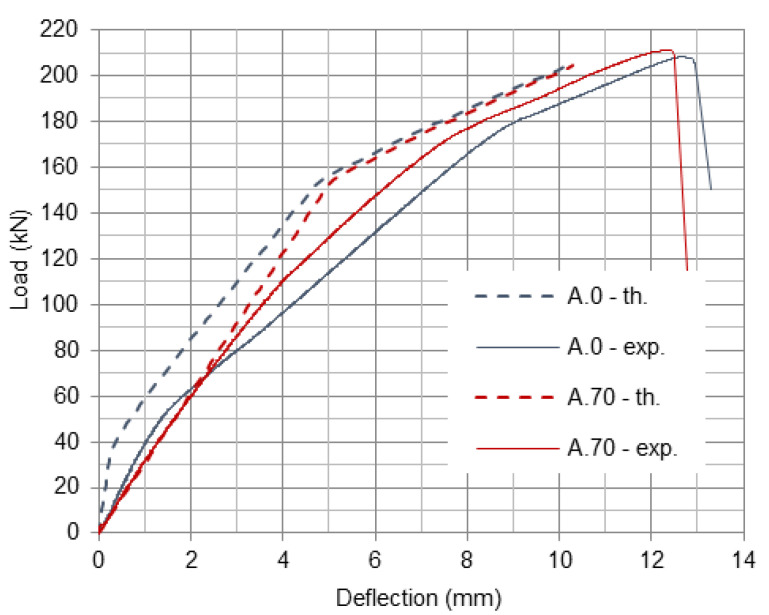
Comparison of theoretical and experimental load-deflection plots for A.0 and A.70 beams.

Deflections of the beams, in the subsequent stages, are calculated on the basis of pre-determined curvatures under the ultimate loads for each stage ($\theta_{cr}$, $\theta_{y}$, $\theta_{u}$), curvature in the place of the load application and geometrical relations between the length of the beam areas in subsequent stages, distance of load application place from the support and beam span [31]. Plots of deflections calculated for beams A.0 and A.70 are presented in Figure 11 and compared with experimental plots. It can be seen that analytical curves have a steeper slope, this indicates that in the theory beams are stiffer than in the experiment. However, the theoretical and experimental curves have a similar shape, which means that analytical model represents well the behavior of the beams without and with preloading. In the A.70 beam, the uncracked stage does not exist because the beam has been cracked during preloading.

### 3.6. Strain Development in CRFP Plates at 0% vs. 70% Preload Levels

**Figure 12:**
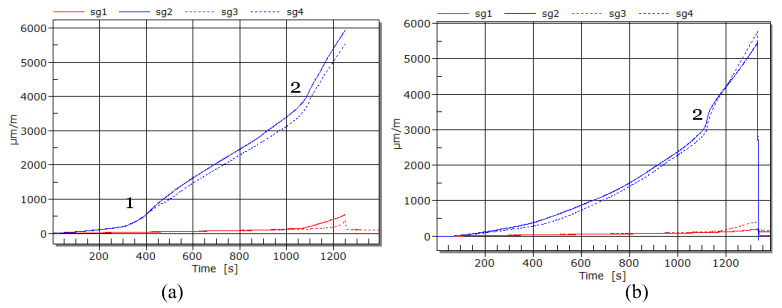
Strain development in CFRP at mid span in (**a**) A.0 (**b**) A.70.

The strain at the middle of the span (i.e., sg2 and sg4) in CFRP plates was monitored during the testing of strengthened beams with different preload levels. Figure 12 shows strain development at 0% preload (i.e., in A.0) and 70% (i.e., in A.70) preload levels. At a 0% preload level, it can be seen that the gradient change with time is similar to load vs. deflection behavior. When the preload level is 70%, two gradients can clearly be seen in the strain vs. time diagram. Moreover, it could be seen that a 7% (see Table 5) decrease of the average strain in 70% preload level compared with the 0% preload level. To control premature debonding, the calculated CFRP strain limit is 0.006171. The average experimental strain obtained at the mid span of the strengthened material (with 0% preloading) is 0.006178. The experimental strain obtained at middle of the span is very close to the calculated value according CNR standard [30] (0.1% difference). For comparison, the recommended strain limits in ACI standard [32] is 0.007159 and in fib guidelines [29] is in range 0.0065–0.0085. Moreover, it has also been reported in the literature that, in most of the cases, theoretical strain limits are much higher than the experimental results [33].

### 3.7. Verification of the Load vs. Deflection Behavior Using FEM Simulation

To verify the experimental findings, a nonlinear FEM simulation has been carried out using software called “ATENA” [34] for strengthened beams without any damage. For the numerical simulation, a 3D finite element model was developed using a GiD interface [35]. To capture the real behavior of the concrete, a three-dimensional eight-node hexahedral solid finite element with a mesh size of 25 mm was used. The mesh sizes were decreased beyond 25 mm and it was found that there is a limited influence on the numerical results. The longitudinal reinforcement and stirrups were modeled as discrete bars with 10 mm mesh size. The CFRP were modeled using plane quadrilateral elements of mesh size 10 mm.

**Figure 13:**
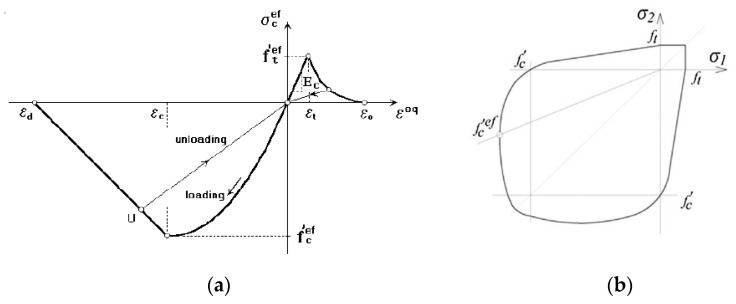
(**a**) Stress-strain law for concrete [35] (**b**) biaxial failure in the Menétrey-Willam hardening/softening model [35].

To model the concrete, the fracture-plastic model: “CC3DNonLinCementitious2” given in ATENA software was used as shown in Figure 13a. The fracture-plastic model combines constitutive models for tensile (fracturing) and compressive (plastic) behavior [35]. The fracture model used in the software is based on the classical orthotropic smeared crack formulation and crack band model. It employs Rankine failure criterion, exponential softening, and it can be used as rotated or fixed crack model. In this analysis, fixed crack model was used. Moreover, the hardening/softening plasticity model is based on Menétrey-Willam failure surface [35]. The biaxial failure law is shown in Figure 13b. The material properties of concrete have been given in Table 6.

**Figure 14:**
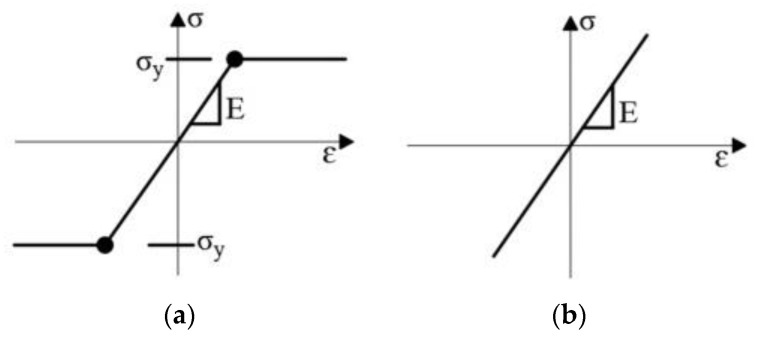
(**a**) Stress-strain law for steel reinforcement; (**b**) stress-strain curve for CFRP plates.

**Figure 15:**
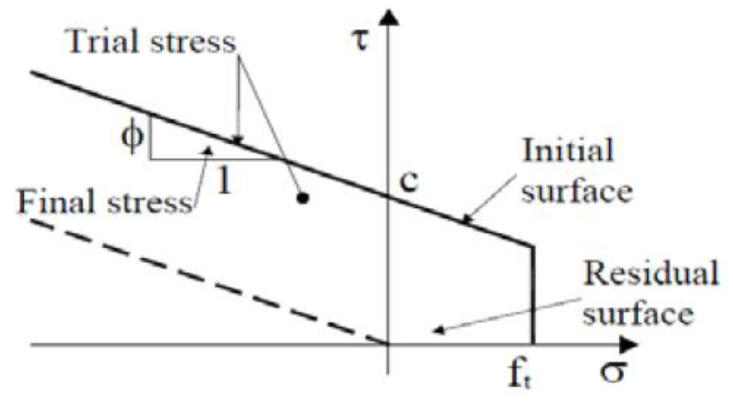
Failure surface for interface element [35].

For modelling the main reinforcement and stirrups, the material model “1D reinforcement” proposed by ATENA software was used. The material model consisted of elastic-plastic material with characteristics yield strength of 500 MP and modulus of elasticity of 200 GPa and the stress-strain curve is given in Figure 14a. The steel plates at the load application points were modelled as a linear elastic material using “CC3D ElasticIsotropic”. The CFRP plate was modelled as an elastic material with mean tensile modulus of 170 GPa and mean tensile strength of 3100 Mpa and the stress-strain curve was given in Figure 14b. The “CC3Dinterface” material type in ATENA software was used to model contact between CFRP plates and the concrete beam. The interface material is based on the Mohr–Coulomb criterion with tension cut off as given in Figure 15. The constitutive relation is given in terms of tractions on the interface planes and relative sliding and opening displacements as given in [35,36]. The normal and tangential stiffness of the interface material have been assumed as 1.34 × 10^7^ MN/m^3^ which gave good agreement between the finite element model and the test results. The cohesion strength of the interface material has been 6 MPa and friction co-efficient has been 0.3.

**Figure 16:**
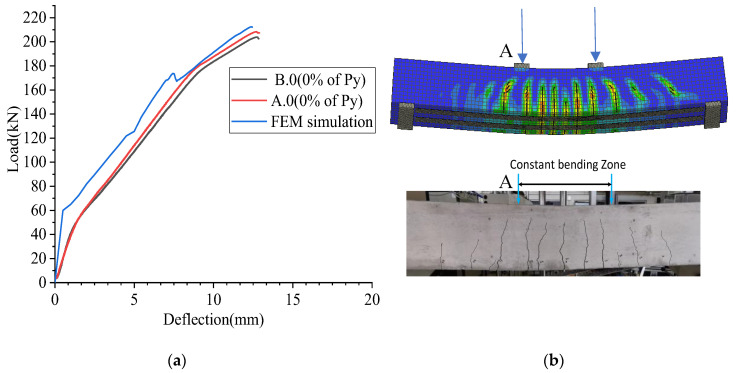
(**a**) Load vs. deflection (**b**) crack formation at 212 kN load during FEM simulation and cracks formed in B.0 beam.

The load was applied as a displacement-controlled method, by giving the displacement in 0.1 mm incremental steps to two load application points. The deflection (i.e., at “A”) and the load at load application points were monitored similar to the experimental testing. The solution of the numerical model was based on the Newton–Raphson method. The results show that ultimate load of the FEM simulation is 212 kN when the deflection is 12.5 mm as given in Figure 16a. This gives good agreement with the failure load of the A.0 beam is 208 kN when the deflection is 12.8 mm. Moreover, five to six cracks have been observed within the constant bending zone during the testing of Beam A.0 and B.0. A similar crack pattern has been observed during the FEM simulation as given in Figure 16b.

## 4. Conclusions

Experimental and numerical investigations have been carried out to determine the failure mechanism and the potential of capacity enhancement that can be attained by externally bonded CFRP.

Preload levels of 0%, 30%, 50% and 70% of Py on the ultimate moment capacity, tangent stiffness, and strain development in CFRP plates, as well as the cracking behavior when a reinforcement configuration of 3Ø12 bars is used on the tensioned side of the beam were considered. Based on the results of the experimental program, analytical procedure, and Finite Element Method (FEM) simulation, the following conclusions have been drawn.

- The first crack formation is delayed in strengthened beams without preloading, compared to strengthened beams with preload conditions.
- The strengthened beams without preload level (0%) and with preload levels (30%, 50% and 70%) indicated similar failure mode. The failure happened by widening the flexural cracks, leading to breaks in the bond between the CFRP plates and concrete, and then, delamination, starting at middle of the beam and then at the ends of the CRP plates. Hence, the failure mode is similar to IFC debonding, leading to plate end debonding or complete debonding of the plates. In addition, based on the quality of the bond between the CFRP plates and concrete, debonding of two plates in a beam happened simultaneously or one plate prior to the other plate.
- Cover separation is not a dominant failure mode in strengthened beams without a preload condition and 30% preloaded beams. However, when increasing preload level from 50% to 70%, slight cover separation is observed at the proximity of wide crack openings.
- When increasing the preload level from 0% to 70%, during failure, wide diagonal cracks (secondary cracks) occurred.
- The initial cracking stage, post cracking stage and post-yielding stage are clearly observed in strengthened beams without preload levels or a 30% preload level, whereas only a post-yielding stage is clearly observed in the 50% and 70% preload conditions. There is no significant difference in tangent stiffness in 0% preload and 30% preload, during the initial cracking stage. However, the tangent stiffness is reduced when the preload level is increased from 30% to 70%.
- There is no significant decrease in the experimental ultimate bending moment, while increasing preload level from 0% to 70%.
- The experimental ultimate bending moment gives good agreement with the theoretical ultimate bending moment, estimated based on the CFRP strain limit given in CNR standard [30].
- The analytical and FEM simulation load-deflection curves give a good agreement with experimental flexural behavior of CFRP strengthened RC beams.
- A capacity increase between 70% and 80%, compared to the un-strengthened member. The test result demonstrates the vast potential of capacity enhancement that can be attained by externally bonded CFRP.

## Figures and Tables

**Table 1:** Composition of the concrete mixture in one cubic meter.

Material	Quantity (kg m^−3^)
Standard cement FA	309
Sand (0/2 mm) Sand (0/8 mm)	306 909
Gravel(8/16 mm)	675
Silica	11
Fly ash	20
Water	131
Admixtures Dynamon SX-23 Mapeair 25 1:19	2.21 0.34

**Table 2:** Mechanical properties of CFRP plates [20].

CarboDur S512	Mean Value	5% Fractile-Value	95% Fractile-Value
Modulus of Elasticity	170 GPa	165 GPa	180 GPa
Tensile strength	3100 MPa	3000 MPa	3600 MPa
Strain at break	Minimum value: 1.8%		

**Table 3:** Properties of epoxy adhesive [21].

Property	Value
Modules of Elasticity in compression	9600 N/mm^2^ (at 2 °CC)
Tensile Modulus of Elasticity	11,200 N/mm^2^ (+2 °CC)
Shrinkage	0.04%
Thermal coefficient of expansion	2.5 × 10^−5^ pe °C (temperature range: −20 °C to +40 °C)
Tensile strength (7 days curing)	26 N/mm^2^–29 N/mm^2^(+15 °C +35 °C)
Density	1.65 kg/L ± 0.1 kg/L

**Table 4:** Summary of the visual inspection.

	Bond Condition	
Beam	Bond Quality	Comment
A.0, B.0, A.30	Good (No defects detected by visual inspection)	Excess adhesive pressed out on both sides of the plates.
B.30	Generally good bond quality	One location with void in adhesive layer. [d~2 mm]
A.50	Poor bond quality	Four locations with relatively deep voids [d~4–9 mm]
B.50	Potentially compromised bond quality	Three locations with relatively deep voids [d~3–6 mm]
A.70	Generally good bond	Two locations with void in adhesive layer [d~1–2 mm]
B.70	Generally good bond	Two locations with void in adhesive layer [d~2–3 mm]

**Table 5:** Experimental results.

Preload	Beam	P_max.ex_ (kN)	Py (kN)	M_R.exp_ (kN.m)	Increase of M_R.exp_ ^1^ (%)	Decrease of Average M_R.exp_ ^2^ (%)	Max Mid Span Strain (µm/m)	Strain Decrease (%)	$\frac{M_{R.exp}}{M_{R.th}}$
0%_U *	A1	-	120	44 (avg)	-	-	-	-	
	B2	-	117	-	-	-	-	-	
	A2	-	112	-	-	-	-	-	
	B1	-	115	-	-	-	-	-	
0%_S *	A.0	208.4	-	79	78	-	5948	-	1.03
	B.0	203.9	-	77	74		6408	-	1.01
30%_S *	A.30	205.4	-	78	75	0	5731	7%	1.02
	B.30	207.6	-	79	77		-		1.03
50%_S *	A.50	192.4	-	73	64	7	4925	20%	0.95
	B.50	207.8	-	79	77		-		1.03
70%_S *	A.70	211.1	-	80	80	1	5763	7%	1.04
	B.70	199	-	76	70		5610	-	0.99

^1^ ((M_Rd.exp_—average of M_Rd.exp_ in ‘0%_U’))/average of M_Rd.exp_ in ‘0%_U’) * 100. ^2^ ((Average of M_Rd.exp_ in ‘0%_S’—average value of M_Rd.exp_ in ‘S’)/average of M_Rd.exp_ in ‘0%_S’) * 100. * ‘S’ refers to strengthened beams and ‘U’ refers un-strengthened beams.

**Table 6:** Properties of concrete.

Properties	Value
Mean cylindrical compressive strength (*f_c_*) (MPa)	49
Modulus of elasticity (*E_c_*) (GPa)	35
Poisson’s ratio (*υ*)	0.2
Mean tensile strength (*f_t_*) (MPa)	3.3
Fracture energy (*G_f_*) (N/m)	147
Fixed crack	1
Plastic strain (*εcp*)	0.0009528
Onset of crushing (*fcu*) (MPa)	7.35
Critical compressive displacement (wd) (m)	0.0005
Density (*γ*) (MN/m^3^)	0.024
Thermal expansion (*α*) (C^0–1^)	0.000012

## Data Availability

All data generated or analyzed during this study are included in this published article.
